# Detecting Binocular Diplopia in Orbital Floor Blowout Fractures: Superiority of the Orthoptic Approach

**DOI:** 10.3390/medicina57090989

**Published:** 2021-09-19

**Authors:** Juraj Timkovic, Jiri Stransky, Petr Handlos, Jaroslav Janosek, Hana Tomaskova, Jan Stembirek

**Affiliations:** 1 Clinic of Ophthalmology, University Hospital Ostrava, 17. Listopadu 1790/5, 708 52 Ostrava, Czech Republic; timkovic.j@bluepoint.sk; 2 Department of Craniofacial Surgery, Faculty of Medicine, University of Ostrava, 703 00 Ostrava, Czech Republic; jiri.stransky@fno.cz; 3 Clinic of Oral and Maxillofacial Surgery, University Hospital Ostrava, 708 52 Ostrava, Czech Republic; 4 Department of Forensic Medicine, University Hospital Ostrava, 708 52 Ostrava, Czech Republic; petr.handlos@fno.cz; 5 Center for Health Research, Faculty of Medicine, University of Ostrava, 703 00 Ostrava, Czech Republic; janosek@correcta.cz; 6 Department of Epidemiology and Public Health, Faculty of Medicine, University of Ostrava, 703 00 Ostrava, Czech Republic; hana.tomaskova@osu.cz; 7 Laboratory of Molecular Morphogenesis, Institute of Animal Physiology and Genetics CAS, 602 00 Brno, Czech Republic

**Keywords:** diplopia, orthoptic examination, orbital blowout fractures, orbit, maxillofacial surgery, ocular motility disorder

## Abstract

*Background and Objectives:* In patients with orbital floor blowout fracture (OFBF), accurate diagnosis of ocular motility disorder is important for decisions about conservative or surgical therapy. However, the accuracy of the traditional test for detecting binocular diplopia/ocular motility disorder using a moving pencil or finger (hereinafter, “finger test”) has been generally accepted as correct and has not been subject to scrutiny so far. Hence, its accuracy relative to full orthoptic examination is unknown. *Materials and Methods:* In this paper, the results of the “finger test” were compared with those derived from a complex examination by orthoptic tests (considered “true” value in patients with OFBF). *Results:* “Finger test” detected ocular motility disorder in 23% of patients while the full orthoptic examination proved much more efficient, detecting ocular motility disorder in 65% of patients. Lancaster screen test and test with color filters were the most important tests in the battery of the orthoptic tests, capable of identifying 97.7% and 95.3% of patients with ocular motility disorder, respectively. Still, none of the tests were able to correctly detect all patients with ocular motility disorder in itself. *Conclusions:* As the presence of ocular motility disorder/binocular diplopia is an important indication criterion for the surgical solution of the orbital floor blowout fracture, we conclude that a complex orthoptic evaluation should be always performed in these patients.

## 1. Introduction

Binocular diplopia is one of the most common problems arising as a result of orbital floor blowout fractures (OFBF) and, at the same time, an important criterion in deciding whether to treat the patient conservatively or surgically [1,2]. It is, however, necessary to note that binocular diplopia is just one manifestation of the ocular motility disorder (OMD) that can arise as a result of herniation of soft orbital tissues in the defect and that such herniation is, in principle, the cause for referring patients for surgical solution [3,4,5].

Still, in clinical practice, binocular diplopia (not OMD in itself) is considered an important indication criterion for the surgical solution [4]. The most widely used method of diagnosing binocular diplopia is a simple test where the surgeon moves an object (often a pencil or a finger; hence, hereinafter, we will call this method a “finger test”) in front of the patient’s eyes. A full orthoptic examination represents an alternative to this method that is capable, thanks to its complexity, of detecting a wider range of OMDs and of its objective quantification [2,3,6,7,8]; it is, however, not widely used [4]. Surprisingly, even after an extensive search, we have found no valid head-to-head evaluation of these two methods in the literature and for this reason, we have decided to perform this study on the differences in the performance of these two methods in diagnosing binocular diplopia resulting from OFBF.

## 2. Materials and Methods

The study was performed in accordance with the Declaration of Helsinki and approved by the local Ethics Committee. All patients signed informed consent to participate in the study.

Sixty-six patients with orbital floor blowout fracture aged 15–80 with a computed tomography- (CT-) proven OFBF were included in the study. Exclusion criteria were: known pre-existing ocular motility disorder, diplopia, or spatial vision disorder. The binocular diplopia was determined in these patients after a complex clinical examination and exclusion of other trauma to the facial skeleton using two methods: (i) the most widely used examination of ocular motility, i.e., a simple clinical test when the patient followed the surgeon’s finger moving in eight gaze directions (up, down, left, right, and four diagonal directions) and convergence. Hereinafter, we will refer to this examination as a “finger test”, which is typically performed by a maxillofacial surgeon and diplopia is reported by the patient; and (ii) by a complex ophthalmological (evaluation of refraction, near and far vision, intraocular pressure, biomicroscopic examination of the anterior and posterior segments of both eyes) and orthoptic examination (consisting of the “finger test” itself combined with examination of fixation, accommodation and convergence, of binocular spatial functions using color filters, Worth four lights and Bagolini striated glasses tests, the degree of strabismus in prism cover test and by synoptophore, and Lancaster screen test) performed by an ophthalmologist. Both examinations were performed on the same day and never later than two weeks after the injury (sooner if the swelling of soft tissues subsided before that). The combined result of the complex evaluation was used as a reference value (i.e., (our “gold standard” for diplopia)) for subsequent test performance evaluations.

The difference between the results of both tests was evaluated using the chi-square test in the contingency table. Performances of the individual tests relative to the “true” value (i.e., presence of the ocular motility disorder measured as a complex combination of all orthoptic examinations based on the standard methodology of orthoptics) were calculated in Stata v.14 (StataCorp LLC, College Station, TX, USA). The raw data is available from https://doi.org/10.6084/m9.figshare.14538111.v1 (accessed on 10 September 2021).

## 3. Results

The mean age in our patient group was 43 years (SD = 19; median age 42 years, min-max 15–81 years). Out of the 66 patients participating in our study, 45 were male and 21 were female. 31 patients were treated surgically, 35 conservatively. 43 patients (65.2%) with isolated orbital floor blowout fracture had an ocular motility disorder and out of these, 41 suffered from binocular diplopia detected by a complex orthoptic examination.

Thirteen patients in our group were treated conservatively despite having ocular motility disorder. Out of these, 6 (46.1%) had detectable strabismus that were neither on synoptophore nor on the cover test. In 10 of these patients (76.9%), Lancaster screen proved the limitation of ocular motility on the affected side, which was most likely caused by a swelling of the orbital soft tissues that regressed spontaneously over time. Twelve conservatively treated patients with ocular motility disorder had also diplopia but were not surgically treated because, based on the complex orthoptic examination and CT results, the diplopia was, at the time, judged to be a result of soft tissue swelling. In 4 such patients (30.8%), ocular motility disorder detected by orthoptics persisted until the end of the follow-up period; only one patient, however, suffered from diplopia as well and none of these patients complained of problems in everyday life. The one patient with persisting diplopia had binocular diplopia and ocular motility disorder already during the original examination and, due to a suspected herniation of the inferior rectus muscle, was originally referred for surgery. However, as he suffered from severe renal insufficiency, the surgery in general anaesthesia was abandoned. After a partial recovery of the renal functions (2 months later), he was no longer eligible for surgery due to the long period from the injury and a fully developed orbital scarring. The last conservatively treated patient who had an ocular motility disorder but no binocular diplopia did not develop diplopia by the end of the follow-up period. None of the patients in whom orthoptic examination revealed no ocular motility disorder and who were treated conservatively (22 patients) developed any ocular motility disorder by the end of the follow-up period.

All but one of the 31 patients who underwent surgery had an ocular motility disorder diagnosed by the complex orthoptic examination. The single patient who underwent reconstruction of the orbital floor, despite having no signs of an ocular motility disorder, was operated due to the large size of the orbital floor defect (more than half of the orbital floor area). In 19 of the surgically treated patients, the “finger test” revealed no diplopia.

We recorded success (absolute or partial) in 77% of surgically treated patients. Surgery had no effect in 16% (5 patients) in whom minor disorders persisted after surgery (binocular diplopia in one of the eight non-primary gaze directions). In two more patients who underwent surgery (6%), binocular diplopia persisted in the primary position. One of these two patients underwent surgery to address strabism. Nevertheless, additional prism correction in eyeglasses was necessary in both these patients and yielded satisfactory results.

In all, the standard “finger test” revealed only 15 out of 41 patients with binocular diplopia and 15 out of 43 patients with ocular motility disorder. Therefore, while the “finger test” revealed binocular diplopia only in 23% out of 66 patients with OFBF, the complex orthoptic examination detected ocular motility disorder in 65% of such patients (*p* < 0.01). If considering the full orthoptic examination as a reference method, we detected 15 true positives, 2 false positives, 21 true negatives, and 28 false negatives. In other words, the sensitivity of the “finger test” was 34.8% (95% confidence interval 21.5–51.0%), specificity was 91.3% (70.5–98.5%), negative predictive value was only 42.9% (29.1–57.7%), and positive predictive value was 88.2% (62.3–97.9%).

The performance of selected individual tests from the battery of orthoptic tests, i.e., their capability to detect the binocular diplopia revealed by the entire battery, is detailed in Table 1.

## 4. Discussion

Ocular motility disorders resulting from orbital floor blowout fracture are well manageable if a complex diagnostic approach is used. The overall treatment success in our group was high. Out of 34 patients originally referred for conservative treatment, only a minor ocular motility disorder unaffecting the patients in their normal life persisted in 3 patients at the end of the follow-up period. One patient treated conservatively was originally referred for surgery, which could not be performed due to his overall condition; in his case, binocular diplopia persisted. 77% of surgically treated patients healed completely and the remaining patients in whom some problems persisted suffered from injuries so serious that it is highly unlikely that conservative treatment would yield better results than the surgical approach.

The difference between the detection success of the most commonly used method, the “finger test” and full orthoptic examination, is astounding. The “finger test” actually failed to capture 65.1% of patients suffering from ocular motility disorder as a result of the OFBF. Surprisingly, this crucial information has been completely missing in the literature so far.

Of course, one can object that we could not have even expected the good fit of these as binocular diplopia is just a subgroup of ocular motility disorders; however, we must still bear in mind that in this paper, we discuss patients with OFBF in whom ocular motility disorder is likely caused by herniation. Besides, comparing the results of the “finger test” with those of the color filters test (targeting binocular disparity, i.e., the closest test to detecting solely binocular diplopia of the entire orthoptic battery), the difference is still big. The likely reason is that in the early post-injury period, binocular diplopia can be, in the case of the “finger test”, to a major degree masked by the swelling of orbital soft tissues with drooping upper eyelid, which eliminates the picture projected by the respective injured (squinting) eye, and the patient may not realize the binocular diplopia at all in such conditions. The same applies even when performing a full orthoptic examination, especially when binocular diplopia with vertical disparity in the extreme eye positions is concerned (in particular when looking upwards). However, the full orthoptic examination, unlike the “finger test”, facilitates the exact quantification of the ocular motility disorder and of its dynamics over time. Besides, it also allows detection of a pre-existing ocular motility disorder in patients with strabismus and can distinguish between incomitant and concomitant ocular motility disorder.

Lancaster red–green test appears to be the single most important test of the battery of orthoptic tests, as the combined result of both provided outcomes objectively detected diplopia in 97.7% of cases. The only patient in whom this test failed to detect the ocular motility disorder was a patient with the disorder only apparent in the extreme position (i.e., outside the size of the Lancaster screen). Theoretically, a patient’s non-compliance (shift in the head position) can also confound the result. Patients with “false positive” results in the Lancaster screen test suffered from pre-existing strabismus. The second most important test was the test with color filters for binocular diplopia detection, which missed only two patients with ocular motility disorder and yielded no false positives. Still, none of the individual tests were able to accurately detect all patients with an ocular motility disorder resulting from OFBF, which emphasizes the need for performing the full orthoptic examination. Moreover, it is necessary to note that, for example, performing only Lancaster screen and color filters tests could (i) miss the same patients and (ii) provide false positive results (i.e., had we considered Lancaster screen as a proof of herniation and ocular motility disorder associated with orbital floor blowout fracture, we would come to false conclusions).

The revelation that the “finger test” performs so poorly in comparison to orthoptics has great potential implications for practice. As the presence of binocular diplopia indicates that some parts of the ocular motility apparatus are trapped in the fracture and need to be released, binocular diplopia remains one of the widely recognized indication criteria for surgical resolution of OFBF. Our results suggest that more than half of the patients with binocular diplopia after this injury fail to be detected during a conventional examination. Although binocular diplopia (representing an ocular motility disorder) is not the only indication for surgical therapy, failure to detect it because of using an unsuitable method of examination can lead to erroneous decision to treat the patient conservatively. For this reason, we believe that, where available, a full orthoptic examination should always be performed in patients with OFBF, rather than a simple finger test. The primary aim of treatment is to prevent or eliminate the binocular diplopia limiting the patient’s life and orthoptics facilitates the evaluation of the dynamics, as well as of the rehabilitation success of the ocular motility disorder in both conservative (spatial vision exercises, compensation of strabismus using prismatic glasses) and surgical solutions. This recommendation of the use of orthoptics in patients with OFBF is also in accordance with the proposal by Laurentjoye et al. [5].

The limitation of our study is that it is a single center study. Additionally, we should consider that its results are valid only in the indication of orbital floor blowout fracture and cannot be generalized to any possible ocular motility disorder (although it is likely that similar results would be found there as well). Our results, therefore, need to be confirmed by larger, prospective, multicenter studies; nevertheless, the success rate of our treatment indicates that our method of referring the patients to surgical or conservative treatment based on orthoptics is correct and, therefore, supports the notion that orthoptics is superior to the standard “finger test” in determining ocular motility disorder in patients with OFBF.

## 5. Conclusions

We consider the fact that compared to the full orthoptic examination, the “finger test” usually performed for diagnosing ocular motility disorders failed to reveal 65% of cases, to be of the utmost importance and to be a strong reason for advocating full orthoptic examination in these patients.

## Figures and Tables

**Table 1:** The performance of individual tests from the battery of orthoptic examinations in detecting ocular motility disorders resulting from OFBF (complex combined evaluation used as a reference value, results are presented as estimates with 95% confidence intervals in brackets).

Disorder—Method of Measurement	Sensitivity	Specificity	PPV	NPV
Binocular disparity—color filter test	95.3 (84.2–99.4)	100 (85.2–100)	100 (91.4–100)	92.0 (74.0–99.0)
Strabismus—synoptophore	39.5 (25.0–55.6)	87.0 (66.4–97.2)	85.0 (62.1–96.8)	43.5 (28.9–58.9)
Strabismus—prism cover test	46.5 (31.2–62.3)	91.3 (72.0–98.9)	90.9 (70.8–98.9)	47.7 (32.5–63.3)
Bulbus deviation—Lancaster screen	76.7 (61.4–88.2)	91.3 (72.0–98.9)	94.3 (80.8–99.3)	67.7 (48.6–83.3)
Motility insufficiency—Lancaster screen	93.0 (80.9–98.5)	91.3 (72–98.9)	95.2 (83.8–99.4)	87.5 (67.6–97.3)
Lancaster screen combined	97.7 (87.7–99.9)	91.3 (72.0–98.9)	95.5 (84.5–99.4)	95.5 (77.2–99.9)

PPV—positive predictive value; NPV—negative predictive value; Lancaster screen combined—if any abnormality was detected in any of Lancaster parameters, this parameter was considered abnormal.

## Data Availability

The raw data is available from https://doi.org/10.6084/m9.figshare.14538111.v1 (accessed on 10 September 2021).
